# Recent/Childhood Adversities and Mental Disorders Among US Immigrants

**DOI:** 10.3389/fpsyt.2020.573410

**Published:** 2020-11-09

**Authors:** Manuel Cano, David T. Takeuchi

**Affiliations:** ^1^Department of Social Work, University of Texas at San Antonio, San Antonio, TX, United States; ^2^School of Social Work, University of Washington, Seattle, WA, United States; ^3^Department of Sociology, University of Washington, Seattle, WA, United States

**Keywords:** immigrant, adverse childhood experiences, adversities, substance use, mental disorders, co-occurring disorders

## Abstract

Past research documents the heterogeneity in US immigrants, particularly in terms of racial and ethnic categories and specific ethnic subgroups. The present study builds on this research foundation by investigating heterogeneity in immigrants' experiences of adversity, both recent and during childhood, and associations with mental disorders. Data are drawn from 6,131 adult immigrants in the 2012–2013 National Epidemiologic Survey on Alcohol and Related Conditions-III. Prevalence estimates for mental disorders and adversities were calculated overall and by gender. Latent class analysis was utilized to characterize patterns of self-reported experiences of childhood and recent adversities, and multinomial logistic regression established the statistical association between latent class membership and past-year mental disorder outcomes (substance use disorder only, mood/anxiety/trauma disorder only, co-occurring disorder, or no mental disorder). Neglect was the most commonly-reported childhood adversity among immigrant men and women. Prevalence of meeting criteria for a substance use disorder only, or a mood/anxiety/trauma disorder only, varied between men and women, yet no gender differences were observed in prevalence of co-occurring disorders. For latent class analyses, a five-class solution was selected based on fit indices and parsimony. Approximately 10.0% of the sample was categorized in the latent class characterized by severe childhood adversities, while 57.5% was classified in the latent class with low probabilities of reported adversities. The relative risk of meeting criteria for a past-year substance use disorder only (compared to no substance use or mood/anxiety/trauma disorder) was more than three times as high for members of the class with severe childhood adversities (RRR, 3.26; 95% CI, 2.08–5.10), as well as the class with recent employment/financial adversities (RRR, 3.82; 95% CI, 2.36–6.19), compared to the class with low adversities. The relative risk of past-year co-occurring disorders (compared to no disorder) was more than 12 times as high for those in the severe childhood adversities class (RRR, 12.21; 95% CI, 7.06–21.10), compared to the class with low adversities. Findings underscore the importance of considering both recent and childhood adversities when assessing and providing services for US immigrant groups.

## Introduction

Mental disorders exact high societal, economic, and healthcare costs, contributing to lost productivity, disability, and mortality. Although many immigrant groups in the United States (US) evidence lower rates of mental disorders than the US-born (1–6), immigrants comprise a diverse population with varying levels of risk for mental disorders. Many US immigrants are disproportionately exposed to a variety of stressors with the potential to trigger or exacerbate maladaptive coping and psychopathology. As a means to more adequately address the mental health needs of immigrant populations in the US, a deeper understanding is needed of the intricacies in the relationship between various adversities and mental health outcomes, including substance use disorders, mood/anxiety/trauma disorders, and co-occurring conditions. Prior research has underscored the importance of considering both childhood adversities and more recent stressors and adversities when evaluating impacts on mental health (7). The present study, therefore, examines patterns in experiences of childhood and recent adversities among US immigrants, and the connection to mental health outcomes.

## Background

### Heterogeneity of US Immigrant Populations

According to 2018 data, the US is home to ~44.7 million immigrants (8). Beyond the demographic differences between US-born and foreign-born populations, substantial demographic variation is observed within immigrant populations based on a variety of factors. Differences based on region of origin—and specific country in each region—include educational attainment, income, poverty rate, and English proficiency (8, 9). The demographics of immigrant communities also vary based on period of entry to the US, as patterns in immigrant-sending countries shift over time (8).

The varying journeys, circumstances precipitating migration, and contexts of reception in the US add to the diversity of US immigrants, and each of these factors may influence both access to resources and vulnerability to negative outcomes. While many immigrants have immigration documentation, an estimated 23% of US immigrants lack authorization (10), precluding many opportunities for employment and health insurance and often leading to chronic fear of deportation (11). As immigrants live in the US, they balance the practices, values, identifications (12) and norms of their heritage country and local US community. An immigrant who arrived to the US as an infant may differ in many ways from an immigrant who arrived as an older adult; similarly, an immigrant who has lived in the US for a few years may have a very different experience from an immigrant who has resided in the US for decades.

### Vulnerability to Mental Disorders Among US Immigrants

Although a body of research suggests lower rates of many substance use and psychiatric disorders among US immigrants compared to the US-born, risk is increased among US immigrants who have lived in the US for years (13–15). Moreover, some US immigrants are especially vulnerable to experiencing stressors, traumatic events, and adversities that increase susceptibility to psychopathology and negative coping. Many refugees, asylees, and asylum-seekers (16) have experienced a multitude of traumatic stressors (e.g., witnessing or experiencing risk of death or sexual/physical violence, war, or brutality), in the home countries they fled and as part of their journey to reach the US.

Immigrants may also face stressors related to discrimination or marginalization. The detrimental relationship between perceived discrimination and mental health has been documented among immigrants (17) and various racial/ethnic minority groups (18). Experiences or perceptions of discrimination may vary based on many factors, including education, age, race, heritage, length of time living in the United States, level of assimilation to the host culture, and characteristics of the area of settlement (19–23). Stressors may stem from observed or perceived discriminatory words or actions or from an internalization of one's status as a minority, “outsider,” or “other” (24). Immigrants who are undocumented or have undocumented family members may face additional stressors, including fear of deportation and inability to access essential opportunities in employment, education, or healthcare (11). Immigrants with constrained opportunities and low socioeconomic status may also live with the daily stressors that accompany working in a demanding, low-paying job and/or living in a residentially-segregated neighborhood or an area with high poverty or crime and few economic opportunities (25).

Regardless of socioeconomic status, immigrants may also face stressors related to leaving their home, and often family and friends, behind. Immigrants must balance internal or external expectations regarding retaining the culture of their heritage and adapting to the culture of the host community (12). In addition to the variety of stressors or adversities connected to the motives for immigration, the experiences of immigration, and the conceptualization of immigrants in US society, immigrants may experience a variety of adversities and stressors common to all populations, including adverse childhood experiences and stressful life events such as unemployment, homelessness, divorce, or debt.

### Adverse Childhood Experiences, Recent Stressors, and the Connection to Mental Health

Adverse childhood experiences vary in type and severity—including: abuse of various forms; neglect; exposure to tumultuous household conditions; and living with parents or caregivers with unaddressed mental health concerns (26). Compared to those without such experiences, adult individuals who retrospectively indicate having experienced several types of adversity during childhood may be at greater risk of several adverse health outcomes, including poor mental health (e.g., depressed mood, suicide attempts) and problem substance use (e.g., self-identification as problem drinker, past use of illicit drugs, including intravenous use) (27). While a higher number of self-reported adversities seems to be associated with greater risk for various correlated negative outcomes among individuals across low, middle, and high-income countries (26), prior research has identified that experiencing four adversities represents a threshold at which increased risk of disease is notably salient (27).

Adverse childhood experiences have been associated with a collection of potential health outcomes, and these associations appear to vary in strength, yet not in direction. A recent meta-analysis reported that, for individuals with at least four adversities in childhood (compared to those with no adversities), the pooled odds ratios for risk of a given outcome were consistently >1 for every indicator of poor health examined (26), providing strong evidence of an association between adverse childhood experiences and poor health later on in life. Although weak for some physical health indicators, the relationship appears moderate between adverse childhood experiences and internalizing conditions (e.g., anxiety, low life satisfaction, depression) and strong for externalizing conditions (e.g., problematic alcohol use, problematic drug use) (26).

While childhood adversity may increase susceptibility to mental disorders, additional and compounding factors—particularly recent stress—may play a role in precipitating these disorders (28). The connection between stress and mental disorders may be at least partially determined by cognitive mechanisms. Individual differences in cognitive appraisal and coping explain considerable variation in emotion (29), and therefore a part of what is perceived as stressful is individually constructed (30). Comprising responses to changes in health, family/living situations, work, and finances within the past year (28), recent stress has been identified as a strong predictor of depressive and anxiety disorders (31). The number of stressful life events in the prior year has been associated with risk for major depression, anxiety disorders, and PTSD, yet these associations may vary in strength depending on the adversity that individuals experienced during childhood (32). Life events associated with recent stress are also associated with substance use disorders and play a critical role in supporting or hindering remission from these disorders (33).

#### Gender

Past literature documents gender differences in prevalence of specific adverse childhood experiences, as well as differences in the relationships between these experiences and mental health outcomes in adulthood (34, 35). Gender-related variation with respect to the prevalence, expression, and consequences of several mental disorders is well-documented (36). While prevalence of several substance use disorders is higher in men than women, prevalence of several mood disorders is higher among women than men (37). Moreover, some of these gender-related differences in prevalence or odds of mental disorders vary by nativity in certain population groups (38).

#### Among Immigrants

In US immigrant populations, the childhood adversities and stressful life events experienced may have occurred in the immigrant's country of origin or in the US, depending upon the age at time of immigration. According to data from the World Mental Health Surveys, encompassing 51,945 adults in 21 nations, the prevalence of childhood adversities varies between low/lower-middle income countries, high-middle income countries, and high-income countries (39). In a study utilizing data on a nationally-representative sample of US adults, immigrants evidenced higher relative risk of reporting experiencing childhood neglect, compared to the US-born, yet lower relative risk of reporting experiencing childhood physical/emotional abuse, domestic violence, or sexual abuse (40).

#### Co-occurring Disorders

Experiences of childhood trauma (41), and recent stress (42), are also associated with co-occurring disorders. While both substance use disorders and other mental disorders have implications for disability, lost productivity, social and economic costs, and mortality, the co-occurrence of a substance use disorder with another mental disorder is associated with more negative outcomes than one disorder alone. Co-occurring disorders, compared to one mental disorder only, are tied to factors such as social exclusion, homelessness, unemployment, and isolation (43), and are frequently interconnected with chronic health concerns and poverty (44). A vast body of literature has documented poor outcomes associated with co-occurring disorders (45, 46), including: poor employment, family, and social outcomes (47); aggressive or antisocial behavior, criminal history involvement, recidivism, and self-harm (48, 49); relapse of substance use (48) or dependence (50, 51); and unintentional overdose (52) or suicide (48, 49) in various populations.

An estimated 1.1% of immigrants, compared to 3.1% of the US-born, meet criteria for a past-year co-occurring substance use disorder and depressive or anxiety disorder (53). However, variation in the prevalence of co-occurring disorders has been documented between racial/ethnic groups (53, 54), and by age at time of immigration to the United States (55). Prevalence of co-occurring disorders also varies by gender. Hispanic immigrant women with co-occurring disorders have been identified as a concerning (albeit relatively small) subgroup with elevated risk factors and early onset of psychiatric disorders (56).

## The Present Study

The present study aims to examine some of the heterogeneity in experiences of adversities (both recent and in childhood) and associated mental health outcomes in the diverse US adult immigrant population. A rich body of literature has examined mental health outcomes in US immigrants, using a variety of methods, including latent class analysis. The present study builds on this foundation by using latent class analysis to not only examine immigrants' experiences of childhood adversities (40) but also recent adversities, extending analyses of the interplay of recent and childhood adversities in specific populations [such as female veterans (57), or incarcerated adults (58)] to US immigrant populations. Finally, the study includes both substance use disorders and psychiatric disorders, as well as co-occurring disorders. Co-occurring disorders have been studied in immigrant populations (53, 55, 56, 59), yet less frequently with the person-centered approach facilitated by latent class analysis. A person-centered approach such as latent class analysis affords the opportunity to examine subgroups based on patterns of experiences (60), rather than subgroups classified solely by social constructs such as race.

Although some US immigrants may experience a variety of stressors and adversities specific to their immigrant background (e.g., fear of deportation, experiences of fleeing war or persecution, experiences of living in refugee camps or immigrant detention facilities), the present study focuses on the childhood adversities and past-year adversities that are commonly identified as factors relevant in the development and course of psychiatric disorders in a variety of populations (26–28, 31). First, the present study examines the sociodemographic profile of US civilian non-institutionalized adult immigrants in 2012–2013. Second, the prevalence of various adversities, both in childhood and during the past year, are computed for immigrants overall and for males and females. Prevalence of mental health outcomes is assessed as the percentage of immigrants meeting criteria for a substance use disorder only, a mood/anxiety/trauma disorder only, or a co-occurring disorder. Next, latent class analysis is used to describe patterns in experiences of adversities in childhood and during the past year. Demographic variables are examined as predictors of latent class membership, and latent class membership is subsequently examined as a predictor of mental health outcomes.

## Methods

This study utilized data from the National Epidemiologic Survey on Alcohol and Related Conditions-III (NESARC-III, 2012–2013), a probability sample with a target population of the civilian, non-institutionalized US adult population living in the 50 states or District of Columbia. Participants in NESARC-III were selected via multistage probability sampling, with counties and census-defined blocks serving as primary and secondary sampling units, respectively. Eligible participants were ages 18 or older at the time of screening, not currently on active military duty. Details about the sample design in NESARC-III are available elsewhere (61).

Data were collected in person, using a “fully structured, computer-assisted diagnostic interview” designed to be administered by lay interviewers (62). Interviewers had a minimum of a high school diploma (or GED), and only certified bilingual interviewers administered the interviews in non-English languages. In addition to English, NESARC-III accommodated five languages: Spanish, Korean, Vietnamese, Cantonese, and Mandarin. Of the full sample in NESARC-III (36,309), about 7.3% of the interviews were conducted in a non-English language (61).

NESARC-III utilized the Alcohol Use Disorder and Associated Disabilities Interview Schedule-5 (AUDADIS-5), a diagnostic interview which aims to assess mental disorders, consistent with the fifth version of the Diagnostic and Statistical Manual of Mental Disorders (36). The procedural validity of assessing mental disorders with the AUDADIS-5 has been reported previously (62, 63). NESARC-III did not assess for all the mental disorders that are included in DSM-5, and mental disorders in NESARC-III are provided categorically (i.e., meets criteria for the disorder vs. does not meet criteria for the disorder).

In the present study, individuals identified as “immigrants” were those who responded “no” to the question: “Were you born in the United States?” NESARC-III categorized participants born in US territories as born outside the US. In the present study, data were excluded from: a) two individuals who indicated that they were not born in the US, yet (in a follow-up question) also indicated that the “United States” was their country of birth; b) 24 individuals who indicated that they were not born in the US, but had a response coded as “unknown” for country of birth. Of these 6,378 respondents, 247 individuals with missing data on any of the other variables utilized in the study (3.8% of the eligible sample) were excluded, yielding an analytic sample of 6,131 individuals.

### Measures

#### Outcome Variable: Mental Outcome

The present study generated a composite variable labeled “mental outcome,” including four mutually-exclusive options: (a) “*substance use only*:” met criteria for past-year drug/alcohol use disorder but no mood/anxiety/ trauma disorder; (b) “*mood/anxiety/trauma only*:” met criteria for past-year mood/anxiety/trauma disorder, but no drug/alcohol use disorder; (c) “*co-occurring:*” met criteria for both drug/alcohol use disorder and a mood/anxiety/trauma disorder in the past year; and (d) “*no mental disorder*:” neither drug/alcohol use disorder nor a mood/anxiety/trauma disorder in the past year. For “substance use,” participants met DSM-5 criteria for alcohol or other substance (i.e., sedative, cannabis, prescription opioid, heroin, cocaine, stimulant [whether prescription or illicit], hallucinogen, inhalant/solvent, club drug, heroin, or other drug excluding nicotine) use disorder within the past year. For “mood/anxiety/trauma,” participants met DSM-5 criteria for a mood (major depressive, dysthymia, bipolar I), anxiety (panic, specific phobia, agoraphobia, social anxiety, or generalized anxiety), or trauma (post-traumatic stress) disorder within the past year.

#### Immigration-Related Variables

*Age at time of arrival* to the US (0–11 years; 12–17 years; and 18 years and over) was computed by subtracting the number of years each participant reported living in the US from their chronological age. *Birth region* categorized participants' country of birth into: Europe and Central Asia; East Asia; South Asia; Southeast Asia and Pacific; Middle East and North Africa; Sub-Saharan Africa; Mexico; Central America; Caribbean; South America; and Canada. These categories were informed by the World Bank's classification (64), and by similarities in the ethnoracial and sociodemographic profiles of the sending countries. For example, rather than grouping Canada and Mexico into a common North America region, Canada and Mexico were examined separately. Supplementary Table A provides a list of the countries included in each region.

#### Recent Adversity

##### Self-reported perceived ethnic discrimination

NESARC-III utilized a modified version of the Experiences of Discrimination (EOD) questionnaire (65) to assess experiences of discrimination due to race/ethnicity (formal scoring instructions for this modified questionnaire are not provided by NESARC-III). NESARC-III's modified questionnaire inquired about the frequency (never, almost never, sometimes, fairly often, and very often) of ethnic discrimination across six settings: (1) in obtaining healthcare/health insurance; (2) in treatment or care; (3) in public, on the street, in stores or restaurants; (4) obtaining a job or housing, admission to a school or vocational program, or in the courts or with police; (5) being called a racist name; (6) verbal or physical abuse or threats. Consistent with other studies (66–68), “never” responses were coded with zero, while any other response (including almost never, sometimes, fairly often, or very often) was coded with one. Finally, an overall dichotomous variable—*self-reported perceived ethnic discrimination*—was computed to indicate ethnic discrimination (yes vs. no) occurring at any frequency and setting in the past year (69).

##### Past-year stressors

An index with 16 life events and stressors was included in NESARC-III, and participants responded whether (yes vs. no) they experienced any of 16 plausible stressors during the prior 12 months. The stressor questions included in NESARC-III comprise an index rather than a scale that measures only one construct (results of principal component factor analysis in the present study's sample indicated that the 16 items were indicators of at least five different constructs). NESARC's index encompasses life changes and transitions (e.g., moving to another residence or changing jobs) as well as financial difficulties, relationship conflicts, and family loss. For the purposes of latent class analysis, selection of variables was guided by the research aims; therefore, only a subset of NESARC's stressors were included in the present study, considering that some of the items in NESARC's index referred to events which are relatively more common (e.g., “trouble with your boss or a coworker”) or which are not inherently negative (e.g., “change jobs, job responsibilities or work hours”; or “have anyone new come to live with you”). Similar to prior studies (70, 71), the following items were included in the present study: (a) Were you fired or laid off from a job? (b) Were you unemployed and looking for a job for more than a month? (c) Have you had so much debt that you had no idea how you were going to repay it? (d) Have you declared bankruptcy? (e) Did you get separated or divorced or break off a steady relationship? (f) Have you at any time been homeless? (g) Did you have serious trouble with the police or the law? These items represent measures related to employment/financial instability, relationship instability, residential instability, and legal instability.

#### Childhood Adversity

NESARC-III utilized a retrospective measure of adversity during childhood, reportedly modified from two standardized instruments (34), the 70-item Childhood Trauma Questionnaire (a valid and reliable retrospective measure of child abuse and neglect) (72) and the Conflict Tactics Scales (a valid and reliable measure of reasoning, verbal aggression, and violence within the family) (73). Questions in NESARC-III's measure are also relatively similar to those appearing in Kaiser Permanente's landmark Adverse Childhood Experiences (ACE) Study (27).

NESARC-III included 29 questions covering maltreatment by parents or caregivers before the age of 18 years, family support, domestic violence, and household members with alcohol, drug, mental health, or legal-related issues. To examine associations with psychiatric disorders, prior research utilizing NESARC-III's measure has selected a varying number of adverse experiences, for example ten experiences (74) or 19 experiences (34). Scoring has also varied, with some studies treating items as polytomous (34) or dichotomous indicators (74).

Considering categories of abuse and household dysfunction in the ACE Study (27), as well as the role of neglect in childhood maltreatment (75), the following nine indicators of childhood adversity (presented by category) were included in the present study; these items were dichotomized, consistent with recommendations in the original Conflict Tactics Scales (73). The full list of questions is available in Appendix A:

a. Neglect: before age 18, respondent was made to do age-inappropriate chores, did not receive essential supplies (e.g., clothes), was not fed, or was not taken to receive needed medical treatment; or before age 10, respondent was left alone or unsupervised.
b. Threatened abuse: before age 18, respondent's parents/caregivers threatened to hit or throw something or physically injure the respondent.
c. Verbal abuse: before age 18, respondent was sworn at, insulted, or told hurtful things by parents/caregivers.
d. Physical abuse: before age 18, respondent's parents/caregivers pushed, shoved, slapped or hit respondent, or hit respondent so hard that marks or bruises were left.
e. Sexual abuse: before age 18, respondent was touched, fondled, made to touch someone else's body sexually without consent or understanding, or respondent experienced sexual intercourse (completed or attempted) without consent or understanding.
f. Exposure to intimate partner violence (IPV): before age 18, respondent's female caregiver was pushed, shoved, kicked, bitten or hit, repeatedly hit, or threatened at knife or gunpoint by a husband or boyfriend.
g. Alcohol or drug misuse in the family: before age 18, respondent lived with parent or other adult household member with drug use or problematic alcohol use.
h. Legal or criminal problems in the family: before age 18, respondent's parent or other adult household member served time in jail or prison.
i. Mental health problems in the family: before age 18, respondent's parent or other adult household member was treated/hospitalized for mental illness, attempted suicide, or died by suicide.

#### Sociodemographic Variables

Consistent with other major epidemiologic studies on the topic (62, 76), the following sociodemographic variables were included in the present study: *gender* (male/female); *age* category (18–29, 30–44, 45–64, and 65 years or over); *race/ethnicity* (Non-Hispanic White, Non-Hispanic Black, Non-Hispanic American Indian/Alaska Native, Non-Hispanic Asian, and Hispanic); *educational attainment* (less than high school, high school or GED [General Education Diploma], some college, and Bachelor's degree or higher); *family income* (0–19,999, 20,000–34,999, 35,000–69,999, and 70,000 or higher, representing the total, combined family income in US dollars within the past year, including income from social service programs); and *marital status* (married or cohabitating; widowed, divorced, or separated; and never married).

### Statistical Analysis

All analyses were computed with Stata/MP 16.0. Descriptive analyses examined frequencies (with unweighted data) and relative frequencies (with weighted data) of sociodemographic characteristics, past-year mental disorders, past-year adversities, and childhood adversities. For percentages and prevalence estimates, 95% confidence intervals (CIs) were computed. Prevalence of past-year mental disorders, past-year adversities, and childhood adversities were stratified by gender, due to documented differences in the experience and expression of distress between men and women (37).

Latent class analysis (LCA) was used as an exploratory approach to characterize patterns in respondents' recent and childhood adversities. The seven past-year adversities and the nine types of childhood adversity were modeled as dichotomous, manifest indicators of the latent-class solutions in a binomial model with the logit link function. For class enumeration, several LCA models were fitted via maximum likelihood (without specifying tolerance for the scaled gradient) and compared with goodness-of-fit statistics in unweighted data.

The set of fit indices used to decide the optimal number of classes included the Akaike Information Criterion (AIC), the Bayesian Information Criterion (BIC), and the Bayes Factor, with greater emphasis on the BIC, “the most commonly used and trusted fit index for model comparison” [(77); p. 445]. Because more than one solution was initially supported, solutions were compared and contrasted with each other, considering parsimony and interpretability. The final class solution was estimated with the *svy* suite of commands to accommodate the complex design in NESARC-III.

Posterior probabilities (78) were computed to: (a) classify respondents into the latent class for which they had the highest probability of membership, given their pattern of responses; and (b) estimate respondents' probability of endorsing a manifest past-year adversity or childhood adversity item, conditional on class membership, class by class. Both a table and a figure with these posterior probabilities were created to accommodate reader preferences, and a matrix table was created to depict average posterior probabilities for the most likely class membership.

Relative frequencies of demographic characteristics were presented for each latent class. As a heuristic method (78), class membership (based on posterior probabilities) was regressed on the sociodemographic characteristics in order to estimate relative probabilities of membership in a given class (conditional on a reference class) for each characteristic (i.e., gender, age, country/region of birth, age at time of arrival to the US, educational attainment, family income, and marital status). Lastly, the variable mental disorder was regressed as an outcome in a model including class membership as a predictor and the sociodemographic characteristics as covariates; predicted probabilities (with marginal effects at the mean) were computed and plotted for each mental disorder outcome. Estimated coefficients and 95% CIs were presented as relative-risk ratios. Because of shared variance and increased collinearity with region of birth, the variable “race” was not included in multivariate analyses that included “birth region.”

## Results

Table 1 presents the sociodemographic characteristics of the sample, as well as the weighted estimates. Nearly half of the immigrants (48.0%; weighted data) were of Hispanic ethnicity, with Mexico accounting for the country of birth of more than a quarter of immigrants. The majority (68.8%) of immigrants had arrived in the US as adults, and most (67.2%) were married or cohabitating.

**Table 1:** Sociodemographic characteristics of adult immigrants in the 2012–2013 National Epidemiologic Survey on Alcohol and Related Conditions-III (*n* = 6,131).

**Characteristic**	**Total^**a**^**	**Percentage^**b**^ (95% CI)**
Sex		
Male	2,763	49.2 (47.7–50.6)
Female	3,368	50.8 (49.4–52.3)
Age, years		
18–29	1,183	18.7 (17.3–20.2)
30–44	2,249	34.3 (32.8–35.8)
45–64	1,994	34.2 (32.4–36.0)
≥65	705	12.8 (11.7–14.1)
Race		
NH White	882	18.8 (17.1–20.7)
NH Black	531	6.6 (5.73–7.56)
NH American Indian	11	0.2 (0.08–0.32)
NH Asian	1,261	26.5 (23.7–29.5)
Hispanic	3,446	48.0 (44.7–51.3)
Region/country of birth		
Europe and Central Asia	493	9.6 (8.2–11.3)
East Asia	285	6.5 (5.4–7.9)
South Asia	502	10.5 (8.9–12.3)
Southeast Asia and Pacific	591	12.9 (11.6–14.3)
Middle East and North Africa	164	3.1 (2.5–3.9)
Sub-Saharan Africa	225	2.9 (2.3–3.7)
Mexico	1,963	26.0 (22.9–29.4)
Central America	537	7.2 (6.4–8.2)
Caribbean	854	12.5 (10.8–14.4)
South America	422	6.8 (5.8–7.9)
Canada	95	2.0 (1.5–2.5)
Age at time of immigration, years		
0–11	1,122	18.6 (17.2–20.0)
12–17	803	12.6 (11.6–13.6)
≥18	4,206	68.8 (67.2–70.4)
Educational attainment		
Less than high school	1,791	26.4 (24.3–28.6)
High school or GED	1,418	21.2 (20.0–22.5)
Some college	1,384	23.1 (21.6–24.7)
≥Bachelor's degree	1,538	29.3 (27.3–31.3)
Family income, $		
0–19,999	1,719	23.4 (22.0–24.9)
20,000–34,999	1,582	22.9 (21.5–24.4)
35,000–69,999	1,580	26.8 (25.3–28.4)
≥70,000	1,250	26.8 (25.1–28.6)
Marital status		
Married/cohabitating	3,702	67.2 (65.6–68.6)
Widowed, divorced, or separated	1,170	14.6 (13.5–15.6)
Never married	1,259	18.3 (16.8–19.9)

a *Unweighted results*.

b *Weighted results*.

*CI, confidence interval; NH, Non-Hispanic; GED, General Education Diploma*.

As presented in Table 2, among adult US immigrants, past-year mood/anxiety/trauma disorders alone were more prevalent than substance use disorders alone. However, among adult immigrant men, substance use disorders alone were most prevalent. The prevalence of substance use disorders alone was nearly three times as high in men, compared to women, while the prevalence of mood/anxiety/trauma disorders alone was more than two times as high in women compared to men.

**Table 2:** Prevalence of select past-year mental disorders, recent adversities, and childhood adversities among adult immigrants in the 2012–2013 National Epidemiologic Survey on Alcohol and Related Conditions-III (*n* = 6,131).

		**Prevalence**^**b**^ **(95% CI)**		
**Characteristic**	**Total^**a**^**	**All**	**Men**	**Women**
Past-year mental disorder				
Substance use only	368	5.9 (5.3–6.7)	8.9 (7.8–10.2)	3.1 (2.4–3.9)
Mood/anxiety/trauma only	787	11.8 (10.9–12.7)	7.5 (6.5–8.6)	16.0 (14.6–17.4)
Co-occurring	164	2.7 (2.2–3.3)	3.1 (2.4–4.0)	2.3 (1.7–3.0)
Recent adversity				
Ethnic discrimination	2,429	37.9 (36.2–39.7)	39.4 (37.0–41.9)	36.5 (34.5–38.6)
Getting fired or laid off	324	4.8 (4.3–5.3)	5.5 (4.7–6.4)	4.1 (3.4–4.9)
Unemployed, seeking work for ≥1 month	943	13.9 (12.9–15.0)	14.0 (12.6–15.5)	13.9 (12.4–15.5)
Separated, divorced	340	4.1 (3.7–4.6)	3.9 (3.2–4.6)	4.4 (3.7–5.2)
Declared bankruptcy or had so much debt	658	9.3 (8.6–10.1)	9.2 (8.1–10.5)	9.4 (8.4–10.4)
Serious trouble with police, the law	56	0.8 (0.6–1.1)	1.3 (0.9–1.9)	0.3 (0.2–0.6)
Homelessness	69	1.0 (0.8–1.3)	1.1 (0.7–1.6)	0.9 (0.6–1.3)
Childhood adversity				
Neglect	2,211	35.2 (33.6–36.9)	38.8 (36.6–41.0)	31.8 (29.8–34.0)
Threatened abuse	1,869	29.8 (28.2–31.5)	32.4 (30.4–34.5)	27.3 (25.2–29.5)
Verbal abuse	1,497	23.7 (22.3–25.2)	25.2 (23.3–27.2)	22.2 (20.5–24.1)
Physical abuse	1,736	27.9 (26.3–29.4)	30.3 (28.1–32.5)	25.5 (23.6–27.6)
Sexual abuse	524	7.7 (6.9–8.5)	5.3 (4.3–6.3)	10.0 (8.9–11.3)
Exposure to intimate partner violence	997	15.0 (13.9–16.1)	14.5 (13.0–16.2)	15.4 (13.9–17.1)
Alcohol or drug misuse in family	947	13.9 (12.9–14.9)	13.7 (12.3–15.2)	14.1 (12.7–15.5)
Legal or criminal problems in family	209	2.9 (2.4–3.3)	3.1 (2.4–3.9)	2.6 (2.1–3.3)
Mental health problems in family	167	2.5 (2.1–2.9)	2.2 (1.6–2.9)	2.8 (2.2–3.4)

a *Unweighted results*.

b *Weighted results*.

*CI, confidence interval*.

The most common past-year adversity among adult immigrants was ethnic discrimination (37.9%), distantly followed by stressors related to unemployment (13.9%) or debt (9.3%). The prevalence of most past-year adversities was relatively similar in men and women, with the exception of “serious trouble with police/the law,” more commonly reported in men. Neglect, threatened abuse, and physical abuse were the most frequently reported childhood adversities (35.2%, 29.8%, and 27.9%, respectively). The most prominent gender difference in childhood adversities was observed with respect to sexual abuse, with 10.0% of women reporting childhood sexual abuse, compared to 5.3% of men. Childhood neglect, threatened abuse, and physical abuse were significantly, yet modestly, higher among men than women.

For the latent classes based on recent and childhood adversities, a five-class solution was selected according to fit indices (available in Table 3), interpretability, and parsimony. The Bayesian Information Criteria (BIC) is considered a preferred fit index and recommends the model with the lowest BIC value or with a lessening decrease in BIC value for each additional class (77). The average latent class probabilities for the most likely class membership, for the five-class solution utilized in the present study, are presented in Table 4. As depicted in Table 4, for the selected five-class solution, all average posterior probabilities of assignment for each corresponding latent class exceeded the recommended cut-off point of 0.70 (79).

**Table 3:** Fit statistics used to evaluate ten latent class model solutions, based on recent and childhood adversity indicators, for adult immigrants in the 2012–2013 National Epidemiologic Survey on Alcohol and Related Conditions-III (*n* = 6,131).

**k**	**−2LL**	**AIC**	**BIC**	**BF**
1	71570.02	71602.03	71709.57	0.00E+00
2	62342.00	62408.00	62629.79	1.2089E−114
3	61669.12	61769.13	62105.18	9.041E−109
4	61023.30	61157.30	61607.62	9.3621E−49
5	60662.58	60828.58	61386.44	1.61E+10
6	60552.60	60754.60	61433.44	1.02954E−19
7	60316.92	60552.91	61346.00	6.94E+57
8	60443.74	60711.74	61612.37	2.98188E−30
9	60150.80	60454.79	61476.40	1.45E+22
10	60113.30	60449.30	61578.45	-

k = number of latent classes; −2LL, (−2) * (log-likelihood); AIC, Akaike Information Criterion; BIC, Bayesian Information Criterion; BF, Bayes Factor (77).

**Table 4:** Average latent class probability of assignment for most likely latent class membership, for the selected five class solution, for adult immigrants in the 2012–2013 National Epidemiologic Survey on Alcohol and Related Conditions-III (*n* = 6,131).

	**Class 1**	**Class 2**	**Class 3**	**Class 4**	**Class 5**
Class 1	**0.88**	0.08	0.16	0.02	0.00
Class 2	0.02	**0.83**	0.02	0.01	0.01
Class 3	0.09	0.05	**0.72**	0.06	0.01
Class 4	0.02	0.02	0.09	**0.82**	0.14
Class 5	0.00	0.01	0.01	0.10	**0.85**

*Figures in bold, across the diagonal, represent the average posterior probability for classification in the given class, for observations classified in the corresponding class. For example, the average posterior probability of membership in Class 1 is 0.88 for all observations classified into Class 1*.

**Figure 1:**
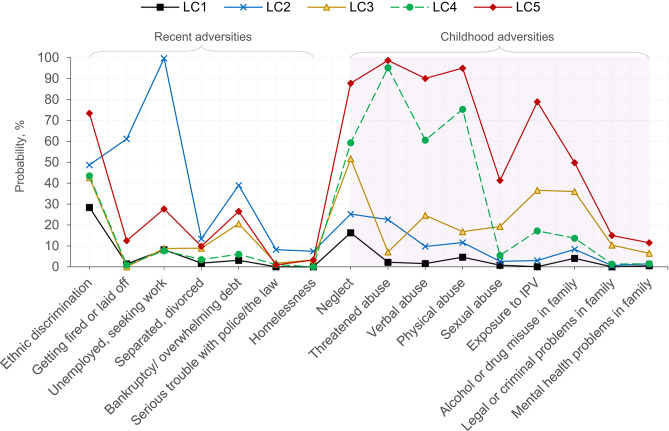
Conditional item probabilities for the selected five-class solution based on recent and childhood adversities for adult immigrants in the 2012–2013 National Epidemiologic Survey on Alcohol and Related Conditions-III (*n* = 6,131). Each marker represents the probability of endorsing an item, given membership in a particular class (e.g., the probability of “Unemployed, seeking work” is 99.7%, given membership in LC2). LC1, low adversities; LC2, recent employment/financial adversities; LC3, elevated childhood neglect/exposure to violence/substance misuse; LC4, childhood physical/psychological abuse; LC5, severe childhood adversities; IPV, intimate partner violence.

Table 5 and Figure 1 present posterior probabilities for class membership, past-year adversities, and childhood adversities for the selected five-class solution. The class with the largest membership (57.5%), Class 1, “low adversities,” was characterized by the lowest recent and childhood adversities. Less than 5% (4.2%) of respondents were classified into class 2, “recent employment/financial adversities,” with the highest levels (compared to any other class) of recent adversities (except ethnic discrimination), including job loss, unemployment, debt, legal/criminal issues, relationship issues, and homelessness. Class 3, “elevated childhood neglect/exposure to violence/substance misuse,” was characterized by above-average childhood neglect, exposure to intimate partner violence, sexual abuse, and family alcohol/drug misuse. Threatened abuse and physical abuse were prominent adversities in class 4, “childhood physical/psychological abuse.” Finally, class 5, “severe childhood adversities,” was distinguished by the highest levels of every type of childhood adversity. Membership in class 5 was also associated with the highest probability (0.73) of reporting past-year ethnic discrimination. Approximately 10% of adult immigrants were predicted to fall under latent class 5.

**Table 5:** Posterior probabilities for the selected five class solution, based on recent and childhood adversity indicators for adult immigrants in the 2012–2013 National Epidemiologic Survey on Alcohol and Related Conditions-III (*n* = 6,131).

	**LC1**	**LC2**	**LC3**	**LC4**	**LC5**	**Overall**
**Class membership probabilities, %**	**57.5**	**4.2**	**10.5**	**17.9**	**10.0**	**100.0**
Recent adversity, %						
Ethnic discrimination	28.4	48.7	42.6	43.5	73.4	37.9
Getting fired or laid off	1.4	61.2	0.0	0.8	12.5	4.8
Unemployed, seeking work for ≥1 month	8.2	99.7	8.8	7.7	27.7	13.9
Separated, divorced	1.8	13.4	8.9	3.5	9.8	4.1
Bankruptcy/ overwhelming debt	3.1	39.0	20.6	6.0	26.5	9.3
Serious trouble with police/the law	0.0	8.2	1.7	1.0	0.9	0.8
Homelessness	0.0	7.4	3.3	0.0	3.3	1.0
Childhood adversity, %						
Neglect	16.3	25.2	51.7	59.3	87.8	35.2
Threatened abuse	2.2	22.6	7.1	95.2	98.7	29.8
Verbal abuse	1.6	9.8	24.6	60.6	90.1	23.7
Physical abuse	4.6	11.6	16.8	75.3	95.0	27.9
Sexual abuse	0.8	2.6	19.3	5.4	41.3	7.7
Exposure to intimate partner violence	0.0	3.0	36.6	17.2	78.9	15.0
Alcohol or drug misuse in family	4.1	8.4	36.0	13.7	49.8	13.9
Legal or criminal problems in family	0.0	0.6	10.4	1.3	15.0	2.9
Mental health problems in family	0.5	1.5	6.5	1.5	11.5	2.5

*Class membership probabilities represent the probability of membership in a latent class. Posterior probabilities for recent and childhood adversity indicators signify the probability of a particular response given membership in that latent class (e.g., the probability of reporting neglect during childhood, given membership in LC1, is 16.3%). As a visual aid, each cell is shaded based on percentage, with darker colors approaching 100%. The “overall” column is provided as a frame of reference for comparison. LC1, low adversities; LC2, recent employment/financial adversities; LC3, elevated childhood neglect/exposure to violence/substance misuse; LC4, childhood physical/psychological abuse; LC5, severe childhood adversities*.

Table 6 presents weighted relative frequencies of demographic characteristics by latent class. Although gender distributions were relatively comparable between classes, males were slightly overrepresented in class 2 (“recent employment/financial adversities”) and class 4 (“childhood physical/psychological abuse”). Class 4 was also the class with the highest proportion of individuals with a bachelor's degree or higher and reported annual family income of $70,000 or higher; immigrants from South Asia and Southeast Asia/Pacific were relatively overrepresented in this class. Nearly three of four individuals categorized in class 1 (“low adversities) had immigrated as an adult, compared to less than three in five individuals categorized in class 5 (“severe childhood adversities”).

**Table 6:** Relative frequencies of demographic characteristics for each latent class, for adult immigrants in the 2012–2013 National Epidemiologic Survey on Alcohol and Related Conditions-III (*n* = 6,131).

**Characteristic**	**Class 1: Low adversities**	**Class 2: Recent employment/ financial adversities**	**Class 3: Elevated childhood neglect/exposure to violence/substance misuse**	**Class 4: Childhood physical/psychological abuse**	**Class 5: Severe childhood adversities**
Sex					
Male	47.1	55.5	47.6	55.8	48.4
Female	52.9	44.5	52.4	44.2	51.6
Age, years					
18–29	18.8	23.3	19.9	17.8	16.2
30–44	31.5	37.1	37.9	36.9	40.3
45–64	33.5	37.3	31.8	35.2	37.7
≥65	16.1	2.3	10.4	10.1	5.8
Region/country of birth					
Europe and Central Asia	11.5	10.2	6.5	7.9	5.1
East Asia	7.3	6.6	3.2	7.3	3.9
South Asia	10.7	6.4	9.8	12.2	8.5
Southeast Asia and Pacific	12.8	6.4	12.2	16.3	10.3
Middle East and North Africa	3.3	1.7	2.4	3.4	3.1
Sub-Saharan Africa	2.5	5.4	4.2	3.3	2.4
Mexico	24.8	29.0	31.8	21.2	34.6
Central America	6.3	8.2	9.3	7.5	9.5
Caribbean	12.4	18.2	13.1	11.1	12.9
South America	6.8	7.6	6.0	7.4	5.7
Canada	1.7	0.4	1.5	2.4	4.0
Age at time of arrival, years					
0–11	14.5	19.1	21.5	25.7	26.1
12–17	11.7	16.6	14.1	11.9	15.6
≥18	73.8	64.3	64.3	62.4	58.2
Educational attainment					
Less than high school	26.8	28.3	29.9	20.8	29.7
High school or GED	21.6	27.0	22.8	18.4	19.9
Some college	21.7	22.7	23.5	24.8	27.5
≥Bachelor's degree	29.8	22.0	23.8	36.0	22.9
Family income, $					
0–19,999	23.4	36.2	25.1	18.2	25.4
20,000–34,999	23.2	25.6	24.1	19.4	25.0
35,000–69,999	25.5	27.0	28.5	29.4	28.3
≥70,000	27.9	11.2	22.3	33.0	21.2
Marital status					
Married	68.4	62.4	61.2	68.9	64.8
Widowed, divorced, or separated	13.8	15.0	19.4	12.6	17.1
Never married	17.8	22.6	19.4	18.5	18.1

*Weighted results from latent class analysis for the selected five class solution, based on recent and childhood adversity indicators. GED, general education diploma*.

Weighted results of the regression of latent class membership on demographic characteristics are presented in Table 7. The probability of membership in the class with the most recent employment/financial adversities (class 2), relative to class 1 (“low adversities”), was lower for females (compared to males) and ages 65 and older (compared to ages 18–29) but higher for individuals from Sub-Saharan Africa or the Caribbean (compared to Europe/Central Asia), individuals who immigrated to the US as children (compared to as adults), and those with family incomes below $70,000 per year. The probability of membership in the class with most childhood adversities (class 5), relative to class 1, was higher for immigrants born in Mexico, Central America, or Canada (compared to Europe/Central Asia), higher for individuals who immigrated as children (compared to as adults), and higher for individuals in the lowest (compared to the highest) annual family income bracket. Relative probabilities of membership in latent classes 2–5, characterized by various childhood or recent adversities (compared to membership in class 1, with low childhood or recent adversities), were higher for individuals who immigrated to the US as children, especially young children, compared to those who immigrated as adults.

**Table 7:** Results of multinomial logistic regression predicting class membership (relative to Class 1, “low adversities”) from sociodemographic characteristics, for adult immigrants in the 2012–2013 National Epidemiologic Survey on Alcohol and Related Conditions-III (*n* = 6,131).

**Characteristic**	**Class 2: Recent employment/financial adversities RRR (95% CI)**	**Class 3: Elevated childhood neglect/exposure to violence/substance misuse RRR (95% CI)**	**Class 4: Childhood physical/psychological abuse RRR (95% CI)**	**Class 5: Severe childhood adversities RRR (95% CI)**
Sex (male, ref.)				
Female	0.71^*^ **(0.53–0.94)**	0.95 (0.77–1.18)	0.71^***^ **(0.59–0.84)**	0.93 (0.74–1.16)
Age (18–29, ref.)				
30–44	1.33 (0.90–1.96)	1.30 (0.94–1.80)	1.41^*^ **(1.09–1.82)**	1.94^***^ **(1.45–2.59)**
45–64	1.35 (0.92–2.00)	1.06 (0.74–1.53)	1.33 (0.98–1.79)	1.93^***^ **(1.36–2.75)**
≥65	0.15^***^ **(0.06–0.35)**	0.68 (0.43–1.05)	0.84 (0.59–1.19)	0.57^*^ **(0.34–0.94)**
Region/country (Europe and Central Asia, ref.)				
East Asia	1.69 (0.80–3.55)	0.64 (0.38–1.07)	0.62^*^ **(0.41–0.94)**	0.64 (0.39–1.07)
South Asia	2.04 (0.88–4.73)	0.53 (0.28–1.01)	0.78 (0.49–1.24)	0.82 (0.46–1.47)
Southeast Asia and Pacific	1.12 (0.53–2.40)	1.00 (0.62–1.61)	0.94 (0.64–1.39)	1.02 (0.59–1.77)
Middle East and North Africa	0.83 (0.31–2.18)	0.80 (0.43–1.47)	0.83 (0.47–1.47)	1.23 (0.55–2.76)
Sub-Saharan Africa	3.54^**^ **(1.54–8.11)**	1.82^*^ **(1.05–3.16)**	1.16 (0.74–1.81)	1.30 (0.67–2.54)
Mexico	1.51 (0.82–2.76)	1.27 (0.87–1.86)	0.82 (0.59–1.13)	1.68^*^ **(1.13–2.49)**
Central America	1.82 (0.85–3.88)	1.49 (0.96–2.31)	1.13 (0.77–1.66)	1.88^*^ **(1.16–3.05)**
Caribbean	2.28^*^ **(1.22–4.28)**	1.09 (0.73–1.61)	0.82 (0.56–1.21)	1.29 (0.87–1.91)
South America	1.86 (0.91–3.82)	0.94 (0.58–1.52)	0.94 (0.60–1.46)	1.08 (0.68–1.72)
Canada	0.49 (0.06–4.19)	0.95 (0.39–2.32)	1.24 (0.63–2.46)	3.12^**^ **(1.60–6.10)**
Age at time of arrival (≥18, ref)				
0–11	1.78^**^ **(1.28–2.47)**	1.72^***^ **(1.33–2.24)**	2.07^***^ **(1.64–2.62)**	2.56^***^ **(1.99–3.28)**
12–17	1.55^*^ **(1.08–2.23)**	1.25 (0.95–1.63)	1.23 (0.93–1.63)	1.64^**^ **(1.23–2.17)**
Educational attainment (≥Bachelor's degree, ref.)				
Less than high school	0.84 (0.51–1.38)	1.04 (0.73–1.47)	0.72^*^ **(0.54–0.96)**	0.92 (0.66–1.28)
High school or GED	1.01 (0.59–1.76)	0.99 (0.71–1.38)	0.71^*^ **(0.54–0.95)**	0.82 (0.61–1.11)
Some college	0.96 (0.60–1.52)	1.02 (0.76–1.37)	0.85 (0.65–1.13)	1.14 (0.83–1.57)
Family income, $ (≥70,000, ref)				
0–19,999	4.82^***^ **(2.83–8.19)**	1.17 (0.82–1.69)	0.92 (0.67–1.25)	1.58^*^ **(1.10–2.28)**
20,000–34,999	3.21^***^ **(1.77–5.82)**	1.13 (0.81–1.58)	0.94 (0.71–1.26)	1.45 (1.00–2.09)
35,000–69,999	2.80^**^ **(1.58–4.94)**	1.23 (0.91–1.66)	1.14 (0.86–1.50)	1.38 (0.99–1.92)
Marital status (married, ref.)				
Widowed, divorced, or separated	1.27 (0.89–1.81)	1.71^***^ **(1.33–2.19)**	1.09 (0.85–1.39)	1.41^**^ **(1.10–1.80)**
Never married	0.93 (0.62–1.40)	1.06 (0.82–1.37)	1.00 (0.80–1.25)	0.99 (0.78–1.25)

*Weighted results. Abbreviations. RRR, relative risk ratio; ref., reference category; GED, general education diploma. As a visual aid, all statistically significant results, for which p is <0.05, are bolded*.

* *p < 0.05*.

** *p < 0.01*.

*** *p < 0.001*.

Table 8 presents weighted results of multinomial logistic regression predicting mental disorder outcomes from sociodemographic characteristics and latent class membership. Adjusted for latent class membership, the relative probability of meeting criteria for a substance use disorder only (compared to no substance use or mood/anxiety/trauma disorder) was higher for individuals who immigrated as children aged 0–11 (compared to those who immigrated as adults) and lower for females (compared to males), for older age groups (compared to those 18–29), and for immigrants born in East Asia, Middle East/North Africa, Sub-Saharan Africa, Mexico, Central America, the Caribbean, or South America (compared to Europe/Central Asia). The relative probability of meeting criteria for a mood/anxiety/trauma disorder only (compared to no substance use or mood/anxiety/trauma disorder) was lower for immigrants from East Asia or Southeast Asia/ Pacific (compared to Europe/Central Asia) and higher for females (compared to males), individuals aged 45–64 (compared to 18–29), and widowed/divorced/separated (compared to married) individuals. The relative probability of meeting criteria for both a substance use disorder and a mood/anxiety/trauma disorder (compared to no disorder) was higher for individuals who had immigrated as children aged 0–11 (compared to as adults) and lower for individuals aged 45–64 and 65+ (compared to 18–29) and immigrants from Mexico (compared to Europe/Central Asia).

**Table 8:** Results of multinomial logistic regression predicting mental disorders (relative to “no mental disorder”) from sociodemographic characteristics and latent class membership for adult immigrants in the 2012–2013 National Epidemiologic Survey on Alcohol and Related Conditions-III (*n* = 6,131).

**Characteristic**	**Substance use only RRR (95% CI)**	**Mood/anxiety/trauma only RRR (95% CI)**	**Co-occurring RRR (95% CI)**
Sex (male, ref.)			
Female	0.37^***^ **(0.27–0.49)**	2.25^***^ **(1.89–2.68)**	0.72 (0.51–1.02)
Age (18–29, ref.)			
30–44	0.69^*^ **(0.49–0.97)**	1.12 (0.85–1.49)	0.68 (0.40–1.15)
45–64	0.54^**^ **(0.35–0.83)**	1.35^*^ **(1.01–1.80)**	0.35^**^ **(0.19–0.66)**
≥65	0.16^***^ **(0.07–0.38)**	1.04 (0.66–1.64)	0.08^***^ **(0.02–0.27)**
Region/country (Europe and Central Asia, ref.)			
East Asia	0.51^*^ **(0.27–0.97)**	0.65^*^ **(0.43–0.98)**	0.70 (0.30–1.64)
South Asia	0.51 (0.19–1.40)	0.66 (0.39–1.12)	0.30 (0.06–1.47)
Southeast Asia and Pacific	0.60 (0.33–1.11)	0.62^*^ **(0.40–0.96)**	0.77 (0.31–1.91)
Middle East and North Africa	0.27^**^ **(0.11–0.66)**	1.06 (0.60–1.86)	1.30 (0.47–3.57)
Sub-Saharan Africa	0.41^*^ **(0.19–0.90)**	0.62 (0.35–1.09)	0.37 (0.13–1.05)
Mexico	0.51^**^ **(0.31–0.84)**	0.78 (0.57–1.06)	0.31^**^ **(0.14–0.67)**
Central America	0.40^**^ **(0.22–0.74)**	0.91 (0.60–1.38)	0.71 (0.29–1.79)
Caribbean	0.37^**^ **(0.21–0.65)**	0.85 (0.59–1.23)	1.04 (0.47–2.28)
South America	0.51^*^ **(0.28–0.92)**	1.09 (0.71–1.67)	1.37 (0.53–3.55)
Canada	0.77 (0.28–2.07)	1.22 (0.59–2.54)	2.38 (0.94–6.00)
Age at time of arrival (≥18, ref.)			
0–11	2.13^***^ **(1.48–3.06)**	1.23 (0.97–1.56)	2.63^***^ **(1.85–3.73)**
12–17	1.17 (0.76–1.78)	1.15 (0.84–1.56)	0.75 (0.38–1.48)
Educational attainment (≥Bachelor's degree, ref.)			
Less than high school	1.29 (0.77–2.16)	1.27 (0.91–1.77)	1.57 (0.57–4.37)
High school or GED	1.32 (0.82–2.13)	0.90 (0.64–1.27)	1.32 (0.64–2.72)
Some college	1.00 (0.67–1.51)	1.11 (0.80–1.52)	1.30 (0.74–2.26)
Family income, $ (≥70,000, ref.)			
0–19,999	0.80 (0.48–1.32)	1.30 (0.90–1.89)	1.38 (0.58–3.30)
20,000–34,999	1.04 (0.59–1.83)	1.02 (0.72–1.44)	1.02 (0.46–2.25)
35,000–69,999	1.07 (0.67–1.73)	1.17 (0.84–1.63)	1.29 (0.65–2.56)
Marital status (married, ref.)			
Widowed, divorced, or separated	1.31 (0.86–2.00)	1.30^*^ **(1.01–1.66)**	1.49 (0.85–2.60)
Never married	1.96^***^ **(1.43–2.68)**	1.09 (0.84–1.42)	1.57 (0.95–2.59)
Latent Class Membership (Class 1, ref.)			
Class 2: Recent employment/ financial adversities	3.82^***^ **(2.36–6.19)**	2.50^***^ **(1.73–3.61)**	5.58^***^ **(2.63–11.81)**
Class 3: Elevated childhood neglect/ exposure to violence/ substance misuse	2.61^***^ **(1.77–3.86)**	2.47^***^ **(1.84–3.32)**	7.11^***^ **(3.76–13.44)**
Class 4: Childhood physical/ psychological abuse	2.54^***^ **(1.77–3.64)**	2.51^***^ **(1.88–3.34)**	4.27^***^ **(2.39–7.66)**
Class 5: Severe childhood adversities	3.26^***^ **(2.08–5.10)**	5.69^***^ **(4.44–7.30)**	12.21^***^ **(7.06–21.10)**

*Weighted results. RRR, relative risk ratio; ref., reference category; GED, general education diploma; Class 1: “Low adversities.” As a visual aid, all statistically significant results, for which p is <0.05, are bolded*.

* *p < 0.05*.

** *p < 0.01*.

*** *p < 0.001*.

Results (Table 8) indicated that the relative probabilities of meeting criteria for a substance use disorder only, mood/anxiety/trauma disorder only, or a co-occurring disorder were elevated for all of the latent classes with recent or childhood adversities (classes 2–5), compared to latent class 1 with “low adversities.” Compared to class 1, relative probabilities of meeting criteria for a substance use disorder only (compared to no disorder) were comparable between the class with “recent employment/financial adversities” (class 2; RRR, 3.82; 95% CI, 2.36–6.19) and the class with the “severe childhood adversities” (class 5; RRR, 3.26; 95% CI, 2.08–5.10). However, the class with “severe childhood adversities” (class 5; relative to class 1) had the most notably elevated probabilities of a mood/anxiety/trauma disorder only or a co-occurring disorder, compared to no disorder. That is, for class 5, the relative probability of meeting criteria for a mood/anxiety/trauma disorder only (compared to no disorder) was more than five times as high compared to class 1, and the relative probability of a co-occurring disorder (compared to no disorder) was more than 12 times as high compared to class 1.

**Figure 2:**
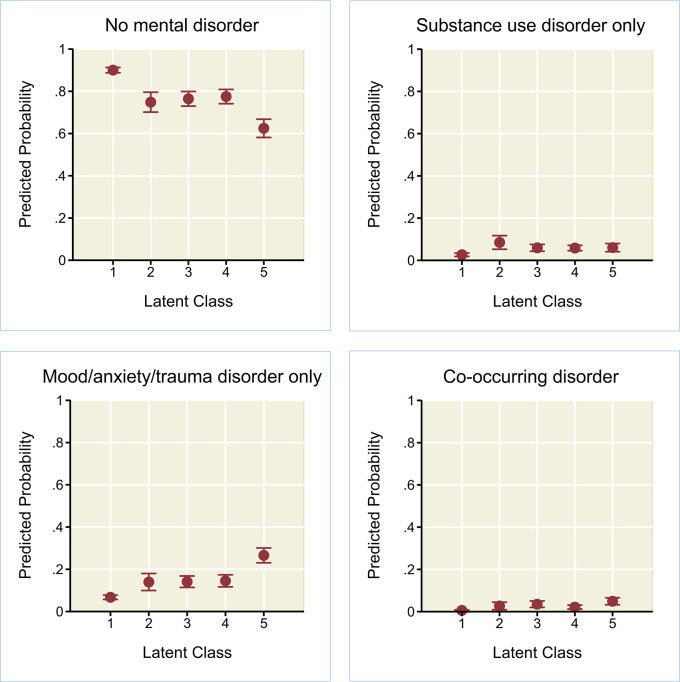
Predicted probabilities (with 95% confidence intervals) of no mental disorder, substance use disorder only, mood/anxiety/trauma disorder only, or co-occurring disorder, by latent class membership for adult immigrants in the 2012–2013 National Epidemiologic Survey on Alcohol and Related Conditions-III (*n* = 6,131). Weighted results of multinomial logistic regression, adjusting for gender, age, country/region of birth, age at time of arrival, educational attainment, family income, and marital status. LC1, low adversities; LC2, recent employment/financial adversities; LC3, elevated childhood neglect/exposure to violence/substance misuse; LC4, childhood physical/psychological abuse; LC5, severe childhood adversities.

Figure 2 depicts predicted probabilities of the four mutually-exclusive mental health outcomes (no mental disorder, substance use disorder only, mood/anxiety/trauma disorder only, or co-occurring disorder) by latent class membership, adjusting for all other variables in the model. For latent class 1 (“low adversities”), the predicted probability of “no mental disorder” was 0.90 (95% CI, 0.89–0.91); probabilities in classes 2–4 were significantly lower than for class 1, yet higher than for class 5 (“severe childhood adversity,” 0.62 [95% CI, 0.58–0.67]). The predicted probability of meeting criteria for a “mood/anxiety/trauma disorder only” was lowest in class 1, higher in classes 2–4, and even higher in class 5 (0.27 [95% CI, 0.23–0.30]). Finally, for co-occurring disorders, predicted probabilities in class 5 (“severe childhood adversities”) were significantly higher than for class 1 (“low adversities”) or class 4 (“childhood physical/psychological abuse”), yet did not significantly differ from class 2 (“recent employment/financial adversities”) or 3 (“elevated childhood neglect/exposure to violence/substance misuse”).

## Discussion

The present study investigated some of the heterogeneity of US immigrants and their experiences of adversity, both during childhood and within the past year. Examining the interplay between childhood and more recent adversities is key to a better understanding of immigrants' needs, not only because of the potential compounded effects of childhood adversity and recent adversity, but also because those currently dealing with particular types of adversity may cope differently based on adversities experienced through the life course.

### Childhood Adversity

The present study found that neglect was the most-commonly reported type of childhood adversity among US immigrant adults, followed by threatened abuse (i.e., threats of violence or harm) and physical abuse. Experiences categorized as neglect may include experiences which respondents perceive as evidencing a lack of care or attention from caregivers, as well as situations in which parents were unable to provide a level of care primarily due to poverty or lack of resources. The ascribed meanings behind these different causes of neglect may be important to consider; it is possible that in low-resource settings such as the countries of origin of some immigrants, or in circumstances of disadvantage and marginalization experienced by some immigrant families in the US, child-rearing norms and access to resources can impact care and result in conditions considered evidence of neglect (40). In a study of the general US adult population, neglect was the most commonly-reported type of childhood adversity, followed by physical abuse (80).

Prevalence of several types of childhood adversities in immigrants in the present study differed from the prevalence estimates documented in US adults overall, consistent with patterns in past research (40). For example, 30.3% of immigrant men and 25.5% of immigrant women in the present study reported experiencing childhood physical abuse, compared to 18.4% of men and 17.5% of women in the general US adult population [from 2011–2014 data (81)]. Conversely, lower proportions of immigrants in the present study reported alcohol or substance misuse or mental health problems in their family during childhood, compared to the general US adult population (81). Overall, the childhood adversities retrospectively reported by immigrants in the present study do not closely mirror the adversities reported by the general US adult population. These differences may be explained by a multitude of factors, including, in some cases, patterns in immigrants' countries of origin. The prevalence of childhood physical abuse is highest in high-middle income countries and lowest in high income countries, while the prevalence of childhood sexual abuse is highest in high income countries (39).

### Perceived Ethnic Discrimination

Of all the adversities examined in the present study (including the seven recent adversities and nine childhood adversities), past-year perceived ethnic discrimination was the adversity with the highest prevalence among US immigrant adults. Past-year perceived ethnic discrimination was reported by 37.9% of immigrant adults, with relatively similar rates in men and women. It is notable that perceived ethnic discrimination was a relatively prominent feature in all the latent classes formed based on adversities, even in the class that was characterized by very low probabilities of other recent or childhood adversities. It is also notable that the latent class with the highest probability of recent adversities (e.g., unemployment, being fired/laid off) was not the class with highest probability of perceived ethnic discrimination; instead, the highest probability of perceived ethnic discrimination was observed in the class characterized by severe childhood adversities, which was also the class with the highest relative risk of co-occurring disorders (compared to no mental disorder).

A large body of research has documented the association between racism or discrimination and poor mental health outcomes (18, 82). Self-reported perceived ethnic discrimination may rely on numerous factors, including exposure to discriminatory acts, cognitive attributions regarding whether experiences reflect ethnic discrimination, recall, and willingness to report these experiences when questioned. Individuals who experienced adversity in childhood are at greater risk for developing mental disorders (26), and those with mental disorders may be prone to greater exposure to certain situations (e.g., seeking mental health services or other supportive services) in which discrimination may be experienced. It is also possible that experiencing childhood adversities contributes to changes in psychopathology, stress sensitization (83, 84), and cognitive attributions (85) that increase the likelihood of attributing negative interactions or events to discrimination. Finally, it is possible that individuals with mental health concerns are more likely to interpret past events as abusive (7), and this pattern may possibly apply both to childhood experiences reported as abuse and recent experiences reported as ethnic discrimination.

### Age at Time of Immigration

Consistent with prior research on the importance of age at time of immigration to the US, the present study highlighted age at time of arrival as a risk factor for poor outcomes. Specifically, risk of meeting criteria for a substance use disorder only or a co-occurring disorder (relative to no disorder) was significantly higher for those who immigrated to the US at ages 0–11, compared to those who immigrated as adults (after controlling for latent class membership and sociodemographic characteristics). Prior research has reported an association between age at the time of immigration to the US and psychiatric disorders, in many (3, 14), yet not all (86) immigrant groups. Individuals who migrate at younger ages are (understandably so) less involved in the decision to migrate (compared to those who migrate at older ages) (14), yet are expected to cope, from an early age, with a multitude of tasks that stem from the process of migration (e.g., ability to reconcile life in two or more cultures) (87). Cumulative adversities (including childhood adversities and recent stressors, among others) are said to reduce gray matter in parts of the brain responsible for regulating cognition, emotion, and behavior, ultimately increasing vulnerability to several psychiatric disorders (88).

Compared to those who immigrated as adults, immigrants who had arrived as children (especially ages 0–11) evidenced higher risk of membership in the latent classes characterized by adversities (relative to membership in the latent class characterized by few childhood or past-year adversities). These differences may be partially explained by the plethora of stressors that are particularly relevant to immigrant youths. Prior to migration, immigrant, refugee, or asylee youths may encounter direct and indirect exposure to interpersonal, institutional, or targeted violence (89), as well as family separation (90). At the time of arrival to the host country, other factors affecting youths, tied to immigration policies, may include separation from parents and increased risk for abuse and exploitation (89). Youths may also encounter a host of issues related not only to adapting/integrating to the host country, but also to the aftermath of exposure to earlier stressors, including internalized distress, particularly among refugee minors (90).

### Substance Use Disorders, Mood/Anxiety/Trauma Disorders, or Co-occurring Disorders

In the present study, the highest risk ratio for the outcome of substance use disorder only (relative to no disorder) was observed in the latent class characterized by recent employment/financial adversities (compared to the class with few recent or childhood adversities). Problematic use of substances can stem from coping responses to stressors, and stressors can hinder remission from drug addiction (33). In a systematic review of risk factors for relapse among individuals with alcohol use disorder, life events involving trauma and stress were identified as factors associated with elevated rates of relapse (50). At the same time, dealing with a mental disorder may also contribute to the formation of particular stressors (or complication of those already existing), by interfering with functional impairment, often observed across various mental disorders. Behaviors surrounding drug seeking, drug use, or addiction can increase individuals' risk of witnessing or experiencing traumatic stressors (e.g., violence, overdose death) (91), or generate stressors in the form of interpersonal conflict, legal consequences, or employment or financial instability.

For the outcomes of mood/anxiety/trauma disorder only or co-occurring disorders (relative to no disorder), the highest risk ratios were observed for the class characterized by severe childhood adversities. The link between adverse childhood experiences and mental disorders or co-occurring disorders has been well-documented (26, 41, 92). Prior research has found that individuals with mental disorders and a history of adverse childhood experiences (compared to those with mental disorders but no history of adverse childhood experiences) may have earlier onset of symptoms, greater severity of symptoms, worse treatment outcomes, and higher risk for comorbidity (93). In the present study, membership in the class with severe childhood adversities (relative to the class with low past-year adversities or childhood adversities) was associated with a twelve-fold elevated risk of meeting criteria for a co-occurring disorder (relative to no disorder). Co-occurring disorders have been tied to a variety of risk factors, negative outcomes, and concerns for diagnosis and treatment success (45–49, 51, 52).

#### Gender Differences

The present study found that the percentage of immigrant adults with co-occurring disorders did not significantly vary by gender; however, other gender differences in mental disorders were identified. The percentage of immigrant adults who met criteria for a substance use disorder only (in the past year) was nearly three times as high among men, compared to women. Conversely, the percentage of immigrant adults who met criteria for a mood/anxiety/trauma disorder only (in the past year) was more than twice as high among women, compared to men. These findings are consistent with prior research documenting gender differences in substance use disorders vs. mood/anxiety disorders (37). The role of sex hormones on cognition and behavior has been identified as a factor representing increased risk of depressive, anxiety, and trauma-related disorders in women compared to men (94). Psychological (e.g., higher tendency toward rumination), interpersonal (e.g., higher rates of violence victimization), and societal factors (e.g., gender discrimination), may also explain the higher prevalence of depression in women, compared to men (94). A multifactorial explanation also exists for gender differences in substance use disorders. Metabolic (in the case of alcohol) and other biological factors explain differences in the effects of substances between men and women (95), potentially also explaining individuals' propensity toward substance use. Beyond biology, social and cultural factors, such as gender-defined roles, influence differential access to substances between men and women (95) and different levels of acceptance of substance use in men and women.

## Limitations

The present study has several limitations. First, the cross-sectional study data confine the results to associations and preclude assertions of temporal precedence. The retrospective nature of the study introduces recall biases, especially relevant for measures of adversities in childhood. A recent meta-analysis documented low agreement between prospective and retrospective reports of childhood maltreatment, concluding that retrospective measures cannot be considered comparable to prospective measures (96); therefore, findings of the present study cannot be generalized to adversities documented via prospective measures. The negative alterations in cognition observed in some mental disorders (36, 85) may influence the degree of perception and report of childhood adversity. The self-report measures utilized also raise concerns of biases, especially for measures related to substance use or other sensitive topics. The mental health outcomes in the study were not assessed by mental health professionals but were determined based on structured interviews administered by lay workers. Although interviewers were available for several languages, not all languages were accommodated in NESARC-III. While NESARC-III oversampled racial/ethnic minority groups, the survey targeted the general population rather than the immigrant population specifically, and it is unclear to what extent subsets of the immigrant population (such as undocumented immigrants or refugees) were represented in NESARC-III's sample.

The questions, and scoring, related to adverse childhood experiences have varied across past studies, and the conceptualization of adverse childhood experiences and cut-off points for the frequency or number of these experiences has also varied in prior research. These considerations hamper direct comparisons between the present study's findings and past results on adverse childhood experiences. The adversities included in the present study do not represent a comprehensive list of adversities; in particular, adversities specifically relevant to immigration experiences (e.g., displacement; war; deportation threats) are not measured in NESARC-III. Moreover, NESARC-III does not provide information on the reasons for immigration or the immigration status of respondents (e.g., refugee, employment visa, undocumented). Thus, an examination of immigration-specific stressors or circumstances of immigration was outside the scope of the present study.

Small numbers of immigrants with mental disorders and immigrant subgroups limited the analyses' statistical power. Since the time of data collection (2012–2013), numerous policy changes have impacted US immigrants (e.g., changes in immigration laws, restrictions on immigrants from certain countries, shifts in access to social programs, increases in anti-immigrant rhetoric, implementation of family separation practices). In light of these changes, the recent adversities and stressors faced by US immigrants may be potentially higher than estimated in this study.

## Implications and Conclusion

Results of the present study highlight some of the heterogeneity in experiences of childhood and recent adversity among US immigrant adults. Findings of the study have implications at clinical, institutional, and policy levels. While demographic characteristics such as nativity, age at the time of immigration, or country of origin constitute critical elements of immigrant health, results of the present study underscore the relevance of childhood and recent adversities (including ethnic/racial discrimination) for screening and intervention. Among service providers, a clearer understanding of immigrants' experiences of adversities may be beneficial for the client-provider relationship. At the same time, the association of age at time of immigration with various experiences of childhood adversities and mental health outcomes highlights the importance of early intervention with immigrant youths living in the US.

At the social services or healthcare systems level, findings regarding co-occurring disorders support the need for integrated treatment networks of addiction and mental health services (97) for US immigrants. The highly elevated risk of co-occurring disorders for immigrants in the latent class characterized by severe childhood adversities suggests that services for immigrants with co-occurring disorders should consider and address the childhood adversities that some immigrants may have experienced. Considering that co-occurring disorders are often associated with the most problematic functional and treatment outcomes, and that immigrants face many barriers to receiving mental health services, specialized services represent an often-unmet need. Finally, at the policy level, the study's findings underscore the need for local, state, and national policies that expand access to mental health services for immigrants. Attending to the mental health needs of immigrants is vital to the health and stability of US society overall.

## Data Availability Statement

The data analyzed in this study is subject to the following licenses/restrictions: Limited access dataset is provided by the NIAAA. Requests to access these datasets should be directed to NIAAA-NESARC-III@mail.nih.gov.

## Ethics Statement

The studies involving human participants were reviewed and approved by University of Texas at San Antonio IRB. Written informed consent for participation was not required for this study in accordance with the national legislation and the institutional requirements.

## Author Contributions

DT conceptualized the study and provided feedback in drafting of the manuscript. MC was responsible for data analyses and drafting of the manuscript. Both authors approved the final manuscript.

## Conflict of Interest

The authors declare that the research was conducted in the absence of any commercial or financial relationships that could be construed as a potential conflict of interest.
