# Supplementary material for: Recent/Childhood Adversities and Mental Disorders Among US Immigrants
Source: Front Psychiatry. 2020 Nov 9;11:573410. doi: 10.3389/fpsyt.2020.573410 (PMC7703683; doi:10.3389/fpsyt.2020.573410)
Supplement: Supplementary file 1 [file Table_1.docx]

| **Supplemental Table 1**  *List of countries of birth of the US immigrant adults in the study, by world region* | |
| --- | --- |
| Region | Country/countries included |
| Europe and Central Asia | Germany, England, Italy, Poland, Russia, France, Ukraine, Spain, Ireland, Turkey, Netherlands, Romania, Bulgaria, Portugal, Hungary, Scotland, Austria, Armenia, Greece, Sweden, Czech Republic, Albania, Denmark, Latvia, Lithuania, Belgium, Moldova, Serbia, Slovakia, Switzerland, Bosnia and Herzegovina, Norway, Belarus, Finland, Georgia, Iceland, Kazakhstan, Macedonia, Slovenia, Turkmenistan, Azerbaijan, Croatia, Cyprus, Estonia, Kyrgyzstan, Uzbekistan, Andorra, Channel Islands, Faeroe Islands, Gibraltar, Greenland, Isle of Man, Liechtenstein, Luxembourg, Monaco, Montenegro, San Marino, Tajikistan, other/unknown Russian, other/unknown European. |
| Sub-Saharan Africa | Angola, Benin, Botswana, Burkina Faso, Cape Verde, Cameroon, Central African Republic, Chad, Comoros, Republic of the Congo, Ivory Coast, Equatorial Guinea, Eritrea, Swaziland, Ethiopia, Gabon, Gambia, Ghana, Guinea, Guinea Bissau, Kenya, Lesotho, Liberia, Madagascar, Malawi, Mali, Mauritania, Mozambique, Namibia, Niger, Nigeria, Rwanda, Sao Tome and Principe, Senegal, Seychelles, Sierra Leone, Somalia, South Africa, Sudan, Tanzania, Togo, Uganda, Zambia, Zimbabwe, Reunion, Mayotte |
| South East Asia and Pacific | Brunei, Cambodia, Indonesia, Laos, Malaysia, Burma/Myanmar, Philippines, Singapore, Thailand, Vietnam, Australia, American Samoa, Micronesia, Guam, Samoa, Tonga, Fiji, New Zealand, New Caledonia, Tuvalu, French Polynesia, Kiribati, Marshall Islands, Palau, Salomon Islands, Papua New Guinea, Vanuatu, Wallis and Futuna, Cook Island, Melanesia, Polynesia, other/unknown Oceania, |
| East Asia | China, Mongolia, North Korea, South Korea, Japan, Taiwan, Hong Kong |
| South Asia | India, Pakistan, Bangladesh, Afghanistan, Bhutan, Nepal, Sri Lanka, Maldives |
| Middle East and Northern Africa | Iran, Iraq, Lebanon, Egypt, Israel, Algeria, Jordan, Saudi Arabia, Kuwait, Morocco, Syria, United Arab Emirates, Tunisia, West Bank, Yemen, Bahrain, Djibouti, Malta, Oman, Gaza, Libya, Qatar, Western Sahara, other/unknown Middle Eastern. |
| Mexico | Mexico |
| Central America | Belize, Costa Rica, El Salvador, Guatemala, Honduras, Nicaragua, Panama |
| Caribbean | Puerto Rico, Dominican Republic, Cuba, Jamaica, Haiti, Trinidad, The Bahamas, Antigua and Barbuda, Saint Martin, Barbados, Dominica, Grenada, Saint Vincent and the Grenadines, Virgin Islands (US), Virgin Islands (British), Aruba, Saint Kitts and Nevis, Saint Lucia, Cayman Islands, Anguilla, Marie Galante, Martinique, Montserrat, Netherlands Antilles, Saint Bartholomew. |
| South America | Argentina, Bolivia, Brazil, Chile, Colombia, Ecuador, Falkland Islands, Guyana, Paraguay, Peru, Suriname, Uruguay, Venezuela, other/unknown South America |
| Canada | Canada |
