# Supplementary material for: Recent/Childhood Adversities and Mental Disorders Among US Immigrants
Source: Front Psychiatry. 2020 Nov 9;11:573410. doi: 10.3389/fpsyt.2020.573410 (PMC7703683; doi:10.3389/fpsyt.2020.573410)
Supplement: Supplementary file 2 [file Data_Sheet_1.docx]

**Appendix A**

**Items for Childhood Adversities from NESARC-III**

**Source:** [**https://www.niaaa.nih.gov/sites/default/files/section%2013_Final_11_17_14.pdf**](https://www.niaaa.nih.gov/sites/default/files/section%2013_Final_11_17_14.pdf)

1. Neglect: response other than “never” to any of the following questions
   1. “Before age 18, how often were you made to do chores that were too difficult or dangerous for someone your age?
   2. How often were you left alone or unsupervised when you were too young to be alone, that is, before you were 10 years old?
   3. Before age 18, how often did you go without things you needed like clothes, shoes or school supplies because a parent or other adult living in your home spent the money on themselves?
   4. Before age 18, how often did a parent or other adult living in your home make you go hungry or not prepare regular meals?
   5. Before age 18, how often did a parent or other adult living in your home ignore or fail to get you medical treatment when you were sick or hurt?”
2. Threatened abuse: response other than “never” to any of the following questions
   1. “Before age 18, how often did a parent or other adult living in your home threaten to hit you or throw something at you, but didn’t do it?
   2. Before age 18, how often did a parent or other adult living in your home act in any other way that made you afraid that you would be physically hurt or injured?”
3. Verbal abuse: response other than “never” to, “Before age 18, how often did a parent or other adult living in your home swear at you, insult you or say hurtful things?”
4. Physical abuse: response other than “never” to any of the following questions
   1. “Before age 18, how often did a parent or other adult living in your home push, grab, shove, slap or hit you?
   2. Before age 18, how often did a parent or other adult living in your home hit you so hard that you had marks or bruises or were injured?”
5. Sexual abuse: response other than “never” to any of the following questions
   1. “Before you were 18 years old, how often did an adult or other person touch or fondle you in a sexual way when you didn’t want them to or when you were too young to know what was happening?
   2. Before you were 18 years old, how often did an adult or other person have you touch their body in a sexual way when you didn’t want to or you were too young to know what was happening?
   3. Before you were 18 years old, how often did an adult or other person attempt to have sexual intercourse with you when you didn’t want them to or you were too young to know what was happening?
   4. Before you were 18 years old, how often did an adult or other person actually have sexual intercourse with you when you didn’t want them to or you were too young to know what was happening?”
6. Exposure to intimate partner violence (IPV): response other than “never” to any of the following questions
   1. “Before age 18, how often did your father, stepfather, foster or adoptive father or mother’s boyfriend do ANY of these things to your mother, stepmother, father’s girlfriend, or your foster or adoptive mother:
      1. Push, grab, slap or throw something at her?
      2. Kick, bite, hit her with a fist, or hit her with something hard?
      3. Repeatedly hit her for at least a few minutes?
      4. Threaten her with a knife or gun or use a knife or gun to hurt her?”
7. Alcohol or drug misuse in the family: affirmative response to any of the following questions
   1. “Before you were 18 years old, was a parent or other adult living in your home a problem drinker or alcoholic?
   2. Before you were 18 years old, did a parent or other adult living in your home have some similar problems with drugs?”
8. Legal or criminal problems in the family: affirmative response to, “Before you were 18 years old, did a parent or other adult living in your home go to jail or prison?”
9. Mental health problems in the family: affirmative response to any of the following questions
   1. “Before you were 18 years old, was a parent or other adult living in your home treated or hospitalized for a mental illness?
   2. Before you were 18 years old, did a parent or other adult living in your home attempt suicide?
   3. Before you were 18 years old, did a parent or other adult living in your home actually commit suicide?”
